# Institutional Context of Pest Management Science in the Global South

**DOI:** 10.3390/plants12244143

**Published:** 2023-12-12

**Authors:** Kris A. G. Wyckhuys, Buyung A. R. Hadi

**Affiliations:** 1 Chrysalis Consulting, Danang 50000, Vietnam; 2 Institute for Plant Protection, China Academy of Agricultural Sciences (CAAS), Beijing 100193, China; 3 School of Biological Sciences, University of Queensland, Saint Lucia 4072, Australia; 4 Food and Agriculture Organization (FAO), 00153 Rome, Italy; buyungasmara@gmail.com

**Keywords:** ecological intensification, agroecology, food system transformation, technological trajectories, plant health, institutional capacity, crop protection, development cooperation

## Abstract

The natural sciences are receiving increasing attention in the Global South. This timely development may help mitigate global change and quicken an envisioned food system transformation. Yet in order to resolve complex issues such as agrochemical pollution, science ideally proceeds along suitable trajectories within appropriate institutional contexts. Here, we employ a systematic literature review to map the nature of inquiry and institutional context of pest management science in 65 low- and middle-income countries published from 2010 to 2020. Despite large inter-country variability, any given country generates an average of 5.9 publications per annum (range 0–45.9) and individual nations such as Brazil, Kenya, Benin, Vietnam, and Turkey engage extensively in regional cooperation. International development partners are prominent scientific actors in West Africa but are commonly outpaced by national institutions and foreign academia in other regions. Transnational institutions such as the CGIAR represent a 1.4-fold higher share of studies on host plant resistance but lag in public interest science disciplines such as biological control. Despite high levels of scientific abstraction, research conducted jointly with development partners shows real yet marginal improvements in incorporating the multiple (social–ecological) layers of the farming system. Added emphasis on integrative system-level approaches and agroecological or biodiversity-driven measures can extend the reach of science to unlock transformative change.

## 1. Introduction

Science influences invention, industry, and enterprise [1,2,3], benefiting the prosperity and societal well-being of nations [4]. National scientific development is influenced by geographic, historical, economic, and social variables [5], casting long legacies and disparities in the scale, quality, and visibility of research worldwide [6]. While Western countries produced the most highly-cited papers in the early 2000s [4], nations in the Global South are fast acquiring critical capacity in the natural science domain. Given their long cycles of poverty, food crises, intercontinental dependencies, and deepening impacts of global change [7,8], this enhanced scientific activity is a promising development.

Interdisciplinary science is imperative to resolving global change and transforming agri-food systems [9,10,11]. The food system, in particular, is a driver and victim of the Anthropocene, and concerted action is needed to keep it within a safe operating space on Earth [12,13]. Indeed, contemporary food systems generate major externalities in terms of land use change, biodiversity loss, carbon emissions, and agrochemical pollution [14,15]. Input-intensive agriculture in particular decouples food production from ecological regulation processes [16] and displays feedback loops with climate change or biodiversity loss [17,18,19]. It is increasingly acknowledged that those issues cannot be solved through reductive science, monodisciplinary ‘silo’ approaches, or so-called techno-fixes [9,16,20,21]. Instead, science needs to account for system complexity and break free of disciplinary boundaries. Yet, this need for interdisciplinarity conflicts with current trends towards diminishing scopes of scientific breadth, so-called ‘gatekeeping’, as well as meager funding [22,23]. To what extent agricultural science in the Global South evades these obstacles and breaches disciplinary boundaries is unknown.

Pesticide-centered crop protection lies at the core of pervasive food system externalities. Since the late 1940s, farmers’ over-reliance on synthetic pesticides has resulted in extensive pollution of environmental matrices, e.g., farmland soil or waterbodies [24], bioaccumulation [25], and declining total factor productivity [26]. This practice impacts human health, triggers biocide resistance development, and weakens ecosystem functioning [27]. The Global South is disproportionately affected by these phenomena, e.g., with low-income countries experiencing a 153% increase in pesticide use over the past decade [28], and climate warming may further exacerbate local pest issues [29]. Also, under current pesticide-intensive management, pests, pathogens, and weeds continue to inflict up to 41% of losses in global food staples such as rice and maize [8]. To remediate the above, scientists have emphasized the role of preventative agro-ecological or biodiversity-driven measures [21,30] and the fortification of ecological regulatory forces through a wholesale redesign of farming systems [19,31,32]. Though scientists in the Global South variably consider more sustainable forms of pest management, such as integrated pest management (IPM), agroecology, and biological control [33], they rarely achieve the envisioned social–ecological outcomes, i.e., concrete reductions in pesticide usage [34]. This failure to act upon pesticide abuse has been linked to deficient policies or inadequate stakeholder engagement, a rapid erosion of public interest science, e.g., academic disciplines such as biological control [35], and an absence of appropriate transnational institutions [36]. Some of these issues can be resolved by drawing upon more holistic ‘systems approaches’ and interdisciplinary science [37,38].

Yet to successfully embark upon interdisciplinary ‘systems’ research, one needs to rethink the overall methodology of the scientific enterprise while accounting for its cognitive context, i.e., the underlying motivations, societal determinants, and delimitations [39]. This involves scientists’ values and world views, power structures, and the internal organization or ‘skeleton’ of public research [40,41,42]. As institutions act as ‘selection devices’ that steer the course of technological change [2], a proper understanding of institutional arrangements and the internal configuration of science priorities is important, especially for public interest disciplines such as agro-ecology or biological control [43,44,45]. Equally, proper institutional alignment with spatiotemporal and functional scales of the focal (agro–eco) system is essential to facilitate a transformative change in farming systems [9,16,46]. Yet until the present, the extent to which (public or private) institutions shape pest management research has received limited attention.

Here, we employ a systematic literature review of 65 developing countries to characterize the overarching institutional structure of pest management science. Following a thorough screening of abstracts, we log the organismal and conceptual foci of the published studies, assess the relative involvement of (public and private; domestic and international) institutions, and employ farming system stratification as an analytical lens [47]. By doing so, we aim to assess the extent to which interdisciplinary, system-centric approaches are used to address pest management issues. Next, we examine how institutional context, economic development, and the degree of inter-country cooperation influence the nature and disciplinary breadth of science. Overall, our work provides an unmatched view of the internal organization of pest management science in the Global South and shines a light on the extent to which this institutional context shapes the scientific enterprise.

## 2. Materials and Methods

We employed a quantitative literature review to characterize pest management science in 65 countries in the Global South (Figure S1). Specifically, we covered West Africa (WA), the Middle East (ME), Southeast Asia (SEA), and Latin America and the Caribbean (LAC) excluding Brazil [48] (Table S1). Certain areas were not covered, e.g., South Asia, the Pacific, Central or Eastern Asia, and numerous countries of sub-Saharan Africa. Per country, development status was inferred from the 2020 Gross Domestic Product (GDP; current USD), as extracted from the World Bank data portal, and Human Development Index (HDI) ranking, as obtained through data portals of the United Nations Development Program (UNDP). Following an in-depth revision of abstracts, we curated the data, categorized published studies, and performed statistical analysis (Figure S1).

First, using Web of Science (WoS), we built an initial corpus of publications covering the 2010–2020 time period; a time frame which permitted assessing the current state of pest management science. Literature queries were defined in order to access publications that addressed arthropod pest management science. As such, our exercise excluded any publications that solely covered plant pathogens or weeds. Per the publication, we extracted information on focal biota, research type, IPM themes, farming system strata, and companion biota from the abstract [48]. Topic searches were conducted using the following WoS search string: TS = ((field OR crop*) AND (pest*) AND country). This string was adapted to each country, by replacing the latter parameter with the exact name of one of the 65 focal countries. By doing so, we obtained published research that was either conducted in each given country or publications that were co-authored by researchers from a selected focal country. Both elements conceivably affect crop protection practices within countries. We queried the WoS Core Collection database (1900–2022) using a University of Queensland staff subscription between 1 August and 15 October 2022.

Next, we individually screened titles and abstracts of all 5924 retrieved studies for relevance. In particular, we removed studies that addressed veterinary or human pests, urban pests except for termites, given their impact on crops, and zoonotic or vector-borne disease vectors such as mosquitoes. On the other hand, we retained publications addressing storage pests because their incidence is often affected by field-level management. We removed any studies that covered pesticide handling, residue detection, (eco-)toxicity, dissipation, or degradation kinetics. We equally excluded any studies that addressed pesticide detection in particular matrices. On the other hand, we retained studies that evaluated the susceptibility of crop-feeding herbivores to pesticidal compounds under laboratory or field conditions. As a final step, we marked any duplicate publications and removed those from specific analyses. As such, we obtained a smaller literature corpus, which was then subject to categorization and statistical analysis (Figure S1). Overall, we used the number of logged publications for a given country as an indicator of its overall research output in pest management science over the study period. To examine whether development status affected pest management science, country-level research output was regressed against HDI rank.

As a next step, we thoroughly screened each publication (or study) within the above literature corpus and classified it into the following categories: focal herbivore or crop, type of research study, IPM thematic areas, farming system variables, and (non-pest) companion biota. We built upon the Indicative Crop Classification (ICC) of the Food and Agriculture Organization (FAO) and categorized the focal crops into 13 different categories. One additional category included studies that either covered multiple crop types or in which the focal crop was not specified. For target herbivores, we recorded the scientific name and taxonomic classification of up to six listed biota. Given that herbivores were often only identified at the genus level, we refer to them as ‘taxa’ irrespective of the exact taxonomic resolution. Studies that either listed more than six herbivore taxa or that left focal herbivores unidentified were treated separately. The following four categories were used for the classification of the research type: laboratory and desktop, reviews, greenhouse and semi-field, or field research. Often, one single publication reported on more than one research type.

Next, we determined whether a given publication covered one or more of eight IPM thematic areas [48,49]: 1. diagnostics and morphology; 2. pest detection, sampling, and monitoring; 3. pest forecasting and prediction; 4. pest biology, ecology, and geographical distribution; 5. preventative non-chemical management; 6. curative non-chemical management; 7. preventative chemical management; and 8. curative chemical management. For field and semi-field studies, we also noted to what extent the research covered the 15 farming system variables. These variables accounted for different facets of a social–ecological farming system at increasing complexity and spatial scale, ranging from an individual seed or plantlet to the entire cropping field, farm and farming landscape, or the associated social system. We equally accounted for the gene, space, and time dimensions of diversification [19]. Similarly, we also recorded whether any of the following companion biota were included: 1. weed or non-crop plant; 2. plant pathogen or disease; 3. non-pest herbivore; 4. non-herbivorous soil-dwelling biota, e.g., detritivore or rhizosphere fauna and flora; 5. pollinator; and 6. biological control agent (BCA). For each field or semi-field study, we thus logged the exact number of system variables and companion biota that were included. Lastly, for field studies, we recorded to what extent the research effort aligned with six hierarchical strata of the farming system: soil, plant, field, farm, landscape, and the social system [47]. This hierarchical stratification approach aims to uncover the relationships in a farming system and use those to describe the nature of scientific inquiry that is needed to bring about change, e.g., agroecological transition [47]. The focal pest and imposed pest management regime were excluded from this stratification exercise. An in-depth description of each of the above variables is provided in [48].

Based upon the author affiliations of each publication, we further ascertained its exact geographical coverage and the extent of inter-country cooperation at a sub-regional or continental scale, i.e., the entire continent of Africa. Similarly, we relied upon author affiliations to log the relative involvement of national institutions from the target country, foreign academia, foreign public or private research entities, and international development partners. The latter category comprised FAO, CGIAR, France’s CIRAD and IRD, CABI, CSIRO, the World Vegetable Center, and icipe. Country-level seats of multinational agrochemical or seed suppliers were categorized as foreign private research entities; insect musea were classified as public research entities; and Chinese academies, USDA, and France’s INRAe were classified as foreign research entities. We equally recorded the involvement of 10 major agrochemical companies. Per country and sub-region, we systematically mapped inter-country cooperation and the relative involvement of different institutions and visualized these interactions with chord diagrams, drawn using the ‘circlize’ package of R 4.0.2 software. In the LAC and WA sub-regions, we equally logged and plotted inter-country cooperation at the continental level, i.e., with other countries outside of the focal sub-region.

Linear regression analysis was used to relate countries’ HDI rank to their overall research output. Chi-square tests were used to detect any geographical differences in the extent of institutional engagement, while One- or Two-way Analysis of Variance (ANOVA) was used to assess whether institutional or geographical context affects the relative coverage of farming system strata. Non-parametric statistics, i.e., Chi-square and Mann–Whitney U tests were also used to assess whether the involvement of development partners relates to (the type and number of) crop or herbivore foci, thematic area, system variables, or companion biota. IBM SPSS Statistics version 29.0 was used for all analyses.

## 3. Results

**Figure 1:**
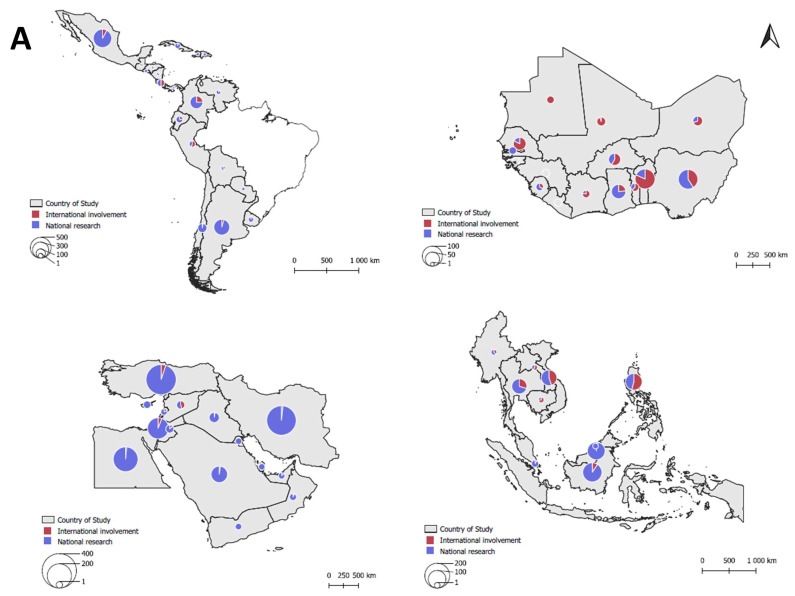

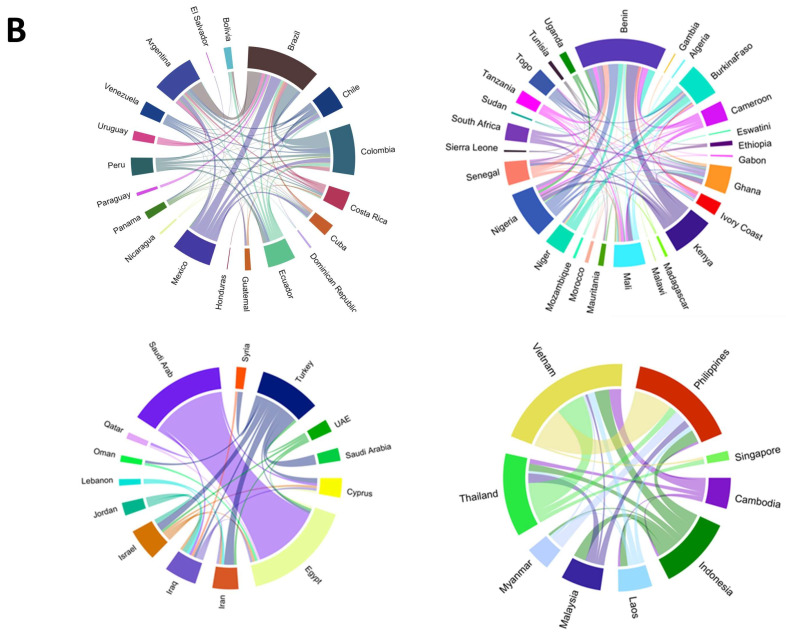
Country-level scientific output and inter-country scientific cooperation in the pest management domain for 65 countries published from 2010 to 2020. Panel (**A**) depicts the total number of peer-reviewed scientific publications (circle size) and the fraction that involves international development partners (red pie section) for countries within LAC, WA, ME, and SEA clockwise from the upper left corner. Countries covered in the literature review are grey-shaded. In panel (**B**), chord diagrams indicate the extent of inter-country cooperation in pest management science per sub-region. Within each diagram, the thickness of an arc between two countries reflects the number of joint publications over the study period. Continent-wide cooperation is shown for LAC (i.e., the inclusion of Brazil) and WA, i.e., through the inclusion of other African nations.

**Figure 2:**
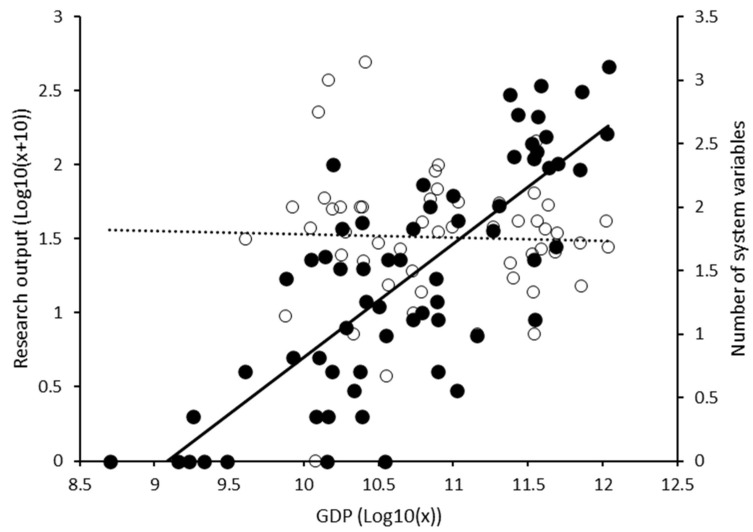
Overall scientific output and extent of system-centric research as related to national economic development for 65 countries in the Global South. Research output (filled dots; primary *Y* axis) is expressed by the total number of peer-reviewed publications per country from 2010 to 2020 and is plotted against countries’ 2020 gross domestic product (GDP). For each country, we equally plot the average number of farming system variables (empty dots; secondary *Y* axis) covered in field studies, capturing the relative extent of system-centric research. The full (vs. dotted) trend line reflects statistically significant linear regression; statistical details are provided in the text. Log-transformed data for GDP and research output are plotted.

After the abstract screening, an initial corpus of 3452 publications was retained. This comprised 614 (SEA); 1362 (LAC); 327 (WA), and 1149 (ME) publications. After the removal of duplicate publications, the final literature corpus comprised 3407 publications. Despite large variability in publication output between the various countries (Figure 1), country-level research output did not differ between sub-regions (ANOVA, F_3,61_ = 0.857, *p* = 0.468). Overall, over the 10-year time frame, an average of 58.8 ± 93.7 (mean ± SD; range 0–459) publications were generated per country. Per sub-region, most publications were logged for Indonesia and Malaysia (SEA; 162 and 138 studies, respectively), Mexico and Argentina (LAC; 459 and 340), Benin and Nigeria (WA; 99 and 95), and Turkey and Iran (ME; 310 and 296). However, 27 countries (41.5%) generate less than one publication per year. Regression analysis shows how country-level research output increases with HDI (ANOVA, F_1,62_ = 7.601, *p* = 0.008) and GDP (F_1,62_ = 98.815, *p* < 0.001, R^2^ = 0.614; Figure 2). Countries such as Benin, Nigeria, or Mexico divert from this pattern, while highly developed nations such as Singapore, United Arab Emirates, Bahrain, or Cyprus feature in two or fewer studies per year. The publications cover a total of 881 (species- or genus-level) herbivore taxa, with 57.4% of studies reporting single taxa and cosmopolitan pests featuring in 67–110 studies per taxon (Figure S2). Most publications address cereal grains (17.6%), annual or perennial fruits (17.3%), and non-starchy vegetables (15.1%).

Various types of institutions are involved in pest management research, i.e., national entities (93.3% studies), foreign academia (38.5%), foreign research centers (25.3%), and development actors (14.6%; Figure S3). CGIAR institutes are the most prominent development partners, involved in 9.2% of global research output. Meanwhile, French institutions or USDA feature in 4.7% and 3.8% of studies, respectively. Lastly, the world’s top 10 agrochemical producers feature in 1.0% of studies. Overall, 8.7% of studies involve inter-country cooperation at the sub-regional level while 21.7% of WA studies engage partners from the remainder of the African continent. Institutional engagement differs geographically for foreign academia (Chi-square *X*^2^ = 137.004, *p* < 0.001), foreign research centers (*X*^2^ = 41.438, *p* < 0.001), and development partners (*X*^2^ = 530.782, *p* < 0.001). National partner involvement in CGIAR-supported studies varies geographically, ranging from 38.4% in LAC to 57.0–65.3% in WA and ME, respectively (*X*^2^ = 9.682, *p* = 0.021). Similarly, the extent of inter-country cooperation at the sub-regional level exhibits geographical differences (*X*^2^ = 74.150, *p* < 0.001). Overall, a respective 7.7%, 18.4%, 26.5%, and 5.7% of studies entail inter-country cooperation in SEA, LAC, WA, and ME. Per sub-region, Vietnam, Brazil, Benin, and Egypt prominently engage in regional cooperation. At the African regional level, Kenya extensively collaborates with WA (Figure 1). Overall, WA and SEA sub-regions receive substantial support from myriad institutions (Figures S3 and S4). For the 30 least-developed countries, development partners engage in a respective 37.8 ± 33.8% (mean ± SD) or 57.1 ± 43.5% of country-level research output or regional cooperative studies. In Benin, Senegal, or Mauritania, development partners are involved in more than 80% of all studies. Development actors equally shape crop and pest foci, e.g., resulting in higher scientific activity on cereal grains (*X*^2^ = 39.271, *p* < 0.001), starchy vegetables (*X*^2^ = 80.234, *p* < 0.001) and cash crops (*X*^2^ = 12.974, *p* < 0.001), and fewer on vegetables (*X*^2^ = 9.420, *p* = 0.002; Figure S2). Similarly, development partners regularly neglect globally relevant pest taxa such as the spider mite *Tetranychus urticae* or potato psyllid *Bactericera cockerelli*.

**Figure 3:**
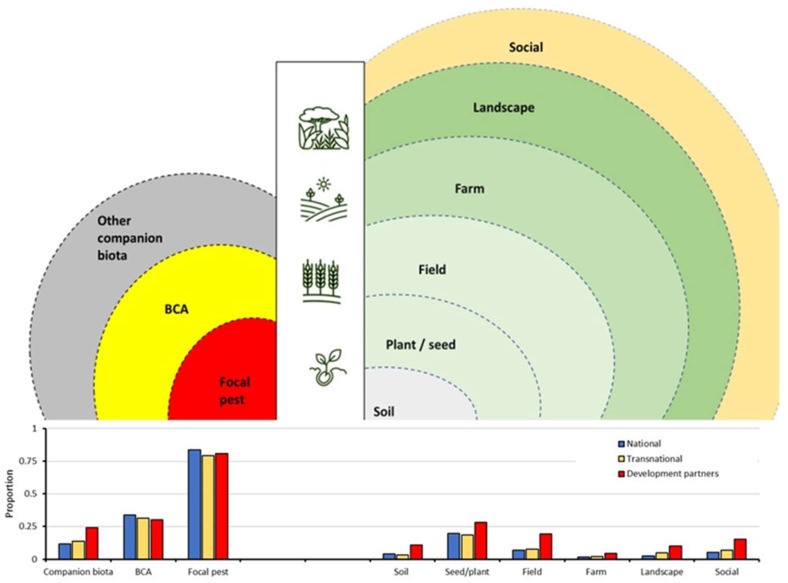
Institutional alignment with the main social–ecological strata and companion biota within a farming system. For 3407 scientific publications published from 2010 to 2020, we contrasted the proportional coverage of different farming system strata (right concentric circles) or biota (left circles) between studies carried out by national institutions (blue), transnational cooperative initiatives (yellow), and work involving development partners (red). For the purpose of visualization, transnational initiatives exclude those featuring development partners. This hierarchical stratification of the farming system unveils the nature of scientific inquiry [47] and its observational context, i.e., delimitations and reductions that are employed [39]. BCAs represent biological control agents.

**Figure 4:**
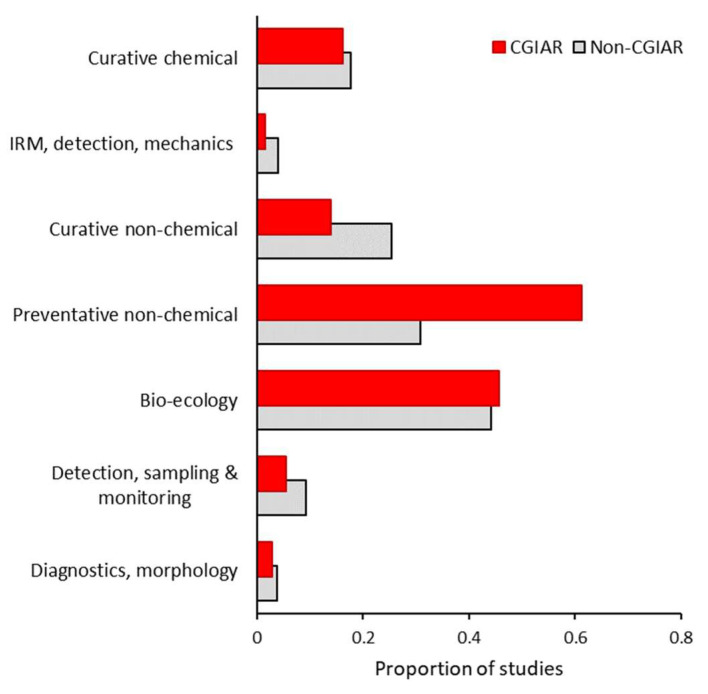
Core thematic areas of CGIAR-supported pest management studies in 65 developing countries. Patterns are shown for 3407 peer-reviewed publications from 2010 to 2020. Specifically, for studies with (n = 315) or without (n = 3092) involvement of the CGIAR, we plot the relative emphasis on seven core pillars of integrated pest management (IPM) as per [49]. The proportion of studies in a given thematic area is plotted, taking into account that some studies address multiple areas. IRM refers to insecticide resistance management.

Overall, 47.9% of publications cover laboratory or desktop research and 49.0% cover field-level studies. In terms of thematic areas, core IPM components such as bio-ecology and preventative and curative non-chemical management are addressed in 44.4%, 33.6%, and 24.4% of studies, respectively. Within the 1832 greenhouse and (semi-)field research studies, 1.8 ± 1.0 (out of 15) farming system variables and 0.6 ± 0.8 (out of 6) companion biota are covered. Among system variables, the target herbivore, crop protection regime, and crop genetics or phenology feature in 81.1%, 29.0%, and 21.0% of studies, respectively. Development partner involvement influences the type of research, thematic areas, system variables, and companion biota (Figure 3). For example, CGIAR-supported studies are less likely to comprise laboratory research (*X*^2^ = 40.776, *p* < 0.001; Figure 4) and more likely to include reviews (*X*^2^ *=* 21.290, *p* < 0.001) or fieldwork (*X*^2^ = 12.827, *p* < 0.001). Equally, CGIAR involvement entails a higher share of studies on preventative non-chemical management (*X*^2^ = 118.527, *p* < 0.001) and a lower share of insecticide resistance management (IRM; *X*^2^ = 4.693, *p* = 0.03), detection and diagnostics (*X*^2^ = 5.231, *p* < 0.001), or curative non-chemical management (*X*^2^ = 20.323, *p* < 0.001) studies. Notably, CGIAR involvement results in a 1.4-fold higher and 75.1% lower share of studies on host plant resistance (HPR; *X*^2^ = 35.727, *p* < 0.001) or botanical insecticides (*X*^2^ = 11.724, *p* < 0.001), respectively. Similarly, engagement from agrochemical companies is reflected in a respective 2.4- and 5.2-fold higher fraction of HPR and IRM studies. Further, the share of CGIAR-backed studies addressing curative non-chemical tactics such as biological control is 45.1% lower than non-CGIAR ones. Lastly, CGIAR involvement in management-centered studies does not affect the number of management types (i.e., curative or preventative; chemical or non-chemical; F_1,2085_ = 0.347, *p* = 0.556).

**Figure 5:**
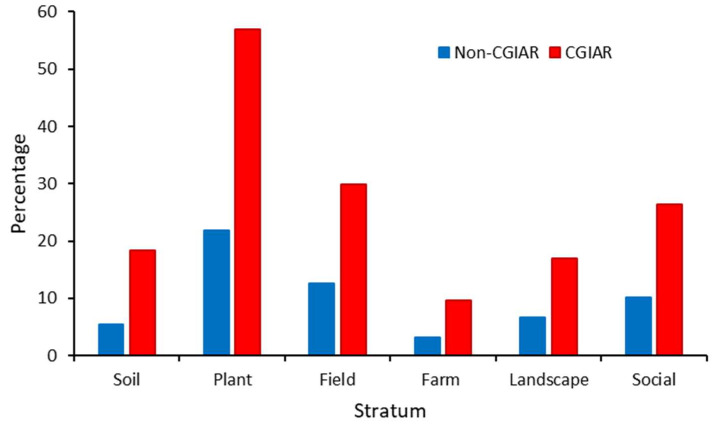
Comparative advantage of development partners in terms of ‘integrative’ pest management and system-centric approaches. Percentual coverage of six (social–ecological) strata is shown for field studies with or without the involvement of the CGIAR specifically. Statistical details are provided in the text.

For management-centered field studies, development partners provide a comparative advantage in terms of the number of system variables (F_1,1043_ = 58.747, *p* < 0.001) and strata (F_1,1043_ = 77.237, *p* < 0.001) but not for integration of management types (F_1,1043_ = 0.565, *p* = 0.452). In all field studies, the number of system variables is affected by geography and CGIAR involvement (ANOVA, geography: F_3,1682_ = 4.768, *p* = 0.001; CGIAR: F_1,1682_ = 31.593, *p* < 0.001; CGIAR × geography: F_3,1682_ = 5.094, *p* < 0.001), but not by GDP (F_1,51_ = 0.054, *p* = 0.818; Figure 2). These effects are not apparent for companion biota. Though consistently more system variables are considered in West Africa (2.0 ± 1.2) and Southeast Asia (1.9 ± 1.1) as compared with the Middle East (1.6 ± 0.9) or Latin America (1.7 ± 0.9), CGIAR-supported field studies cover slightly more system variables (2.3 ± 1.2 vs. 1.7 ± 0.9; out of 15) overall than non-CGIAR ones. CGIAR-supported studies address 46.8–48.3% more system variables for Southeast Asia and Latin America, as compared with 8.3–10.6% for the remaining regions. Notably, the share of CGIAR-supported field studies covering seeds and planting material, crop genetics, soil fertility and plant nutrition, interspecific diversity (over space), or social aspects is a respective 495.1%, 44.6%, 262.8%, 74.2%, or 66.5% higher (*X*^2^ = 20.749, *p* < 0.001; *X*^2^ = 4.122, *p* = 0.042; *X*^2^ = 18.092, *p* < 0.001; *X*^2^ = 12.109, *p* < 0.001; and *X*^2^ = 21.457, *p* < 0.001). CGIAR involvement equally raises proportional coverage of soil, plant, field, farm, landscape, and social strata (*X*^2^ = 13.809, *p* < 0.001; *X*^2^ = 10.452, *p* = 0.001; *X*^2^ = 19.369, *p* < 0.001; *X*^2^ = 4.786, *p* = 0.029; *X*^2^ = 9.949, *p* = 0.002; and *X*^2^ = 21.457, *p* < 0.001) (Figure 5).

## 4. Discussion

In this study, we offer new vistas on pest management science by unveiling the extent to which (a subset of) countries and institutions across the Global South engage in knowledge production. Though nations such as Mexico, Turkey, Indonesia, and Benin annually generate 10–46 publications, 42% of countries generate less than one paper per year. Institutional context strongly modulates focal crop or pest taxa, research type, IPM theme, and system strata. Besides domestic entities, foreign academia and research centers contribute substantially to scientific output. Inter-country cooperation is extensive in Latin America and West Africa, with Brazil and Benin engaging in 41–52% of regional cooperation. Development partners including transnational institutions such as the CGIAR feature in 54% of studies in West Africa, where they build baseline capacity and shape the science agendas of underprivileged nations. Their engagement slightly lowers coverage of ‘top-down’ curative management, pest-centric approaches, and research performed under simplified laboratory settings [48] while furthering interdisciplinary science. However, it does not alter the pursuit of single-factor solutions or techno-fixes. As such, development cooperation offers real, yet marginal improvements in holistic ‘system-centric’ pest management.

Our findings confirm how various countries in the Global South are making sizeable contributions to the scientific knowledge stockpile [5]. Scientific output is most pronounced in Latin America and the Middle East, despite large inter-country variability [50]. Our results show that science in the Middle East has an outspoken domestic profile, while it is strongly affected by transnational cooperation in Latin America and West Africa. In Colombia or Chile, 35–69% of studies entail transnational cooperation at the regional level, far surpassing levels in the UK in the mid-1990s [51]. We further demonstrate how the disciplinary reach (or degree of inclusiveness) of pest management science does not relate to economic prosperity and comprises relatively few natural science domains such as entomology, agriculture, or agronomy. Scientific activity is further limited in Southeast Asian ‘tiger cub’ economies, notwithstanding their rising investment in scientific research [52], broad agricultural base, and historic feats in sustainable pest management [53,54]. This is surprising, as biological control and IPM yielded massive societal benefits across Southeast Asia. For instance, the UN-endorsed IPM farmer field schools during the late 1990s achieved substantial reductions in pesticide usage intensity on millions of hectares across tropical Asia but also in countries such as Peru and Nicaragua [53,55]. Hence, the lasting scientific legacies of these campaigns are few unless those were occluded by methodological shortfalls such as our omission of scientific publications in languages other than English. Also, by using the number of scientific articles as a proxy of research output, we do not account for the (variable) quality of outputs and their concrete impact on improving pest management. Furthermore, given its restricted geographic focus, findings from our study cannot be generalized to all countries of the Global South.

Our work thus shows that scientific output in the pest management domain is substantial, even in less-endowed nations of the Global South. Hence, scientists clearly devote their talents to resolving the intricate challenges of the world’s food system [56]; this need becomes more pressing as modern-day science is less likely to yield ‘paradigm shifts’ [3,57] and thus solve the pesticide-induced externalities that have been apparent since the mid-1900s [25]. Regardless of the absolute volume of publications that is continually generated, our analyses show that the present-day scientific enterprise suffers multiple shortcomings. One of these is that research is oftentimes conducted in simplified observational contexts with near-exclusive pest foci and limited coverage of the various (social–ecological) strata of a farming system. Given the above, we believe that a deliberate course correction in pest management science can be instrumental in achieving a ‘tipping point’ for food system transformation [40,58]. Institutional reform and a careful rerouting of scientific trajectories could thereby raise the pace of incremental change to a point at which science transforms (crop protection) practice.

In addition to capturing overall scientific output, our study shines a light on the institutional investment in and overall (development) support for pest management science in the Global South. Although publication rate per se may not be a robust proxy of pest management achievements in low-income countries, our study shows the ubiquity of public sector actors in knowledge production both at the national level and in international cooperative schemes. Universities from Western nations and emerging economies such as Brazil, Turkey, Kenya, and Mexico engage closely with partners in the Global South. Academia plays a prominent role in global networking and multilateral exchange and hallmark features of disciplines such as biological control, ecosystem services, and soil biology [27,59,60]. Though sporadically perceived as elite enclaves [56], academic institutions engage in the development of foundational knowledge and the trialing of preventative or curative management solutions. Amongst others, they investigate how insect predators or microbiota can either be conserved in farm settings, i.e., know-how that cannot be readily marketed [61] or used as commoditized tools. Given their prominent role in pest management science, non-hierarchical structure, and availability of graduate students [51], academic institutions might be suitable lead operators in a science-driven food system transformation. However, one needs to be mindful that a strong academic engagement is not a panacea for the propagation of public interest science. To avoid disciplines falling victim to the lopsided growth of more prestigious fields [43], institutional structures need to consciously make room for contrasting cognitive routines, i.e., reductionism (molecular science) vs. holism (agroecology), build bridges, and accommodate the related internal organization of science [40].

Our research further uncovers how both national and international scientific endeavors are marked by near-exclusive pest- or crop-centric foci, but they do exhibit varying attention to curative (chemical) management. Both types of institutions prioritize single-factor solutions over integrative, system-level interventions; a practice that has been reported to provoke grand societal challenges such as HPR breakdown, biocide resistance [62], and agrochemical pollution [21,24]. Meanwhile, system-level action through multifaceted non-chemical tactics (e.g., field, farm, and landscape-level diversification bundles) consistently receives marginal attention. This is counterintuitive, as those practices are more likely to deliver ‘win–win’ ecological and economic outcomes on the farm level [63,64]. Given the above, a rekindled sense of social obligation is in order, e.g., in which one may wish to extend physicians’ Hippocratic Oath, i.e., ‘first, do no harm’ into the agriculture domain [65]. If, for example, preventative measures and biodiversity-based tactics effectively defuse pest issues, bolster yield resilience, and keep agriculture within planetary boundaries [13,19,64], academics may no longer need to devote their energies to curative or therapeutic tactics. Thus, a conscious deliberation of the values and goals that drive research should become a basis of mission-oriented endeavors [39] and can prevent science from becoming pointless or paralytic [66]. To ensure that science is motivated by societal vs. mere academic impact, incentive or reward schemes could be adapted [67]. Equally, the boundaries between knowledge and action must be managed to enhance the salience, credibility, and legitimacy of plant health solutions [68]. For example, improving institutional fit through an entwinement with (volunteer) support networks, e.g., in the agro-ecology domain, can help to account for a spatial nesting of pest issues and ecological regulation mechanisms [69]. Our analyses did not detect such close interplay between international and national institutions, although this might be crucial when aiming to promote context-specific vs. universal solutions such as pesticide-based control.

Our study demonstrates how development cooperation only offers marginal, though appreciated, improvements in system-level research. Specifically, development partner engagement results in a mere 2.4%, 31.9%, and 82.4% more management types (out of four), farming system variables (out of 15), or strata (out of six), respectively. Those patterns are outspoken for the most prominent development partner, i.e., CGIAR, which features in 9.2% of total research output across the study countries. Over its 50+ year history, the CGIAR has earned global acclaim for its pioneering agroecology, biological control, and farming systems research [70,71]. Nevertheless, from 2010 to 2020, CGIAR-backed studies presented a moderate advantage in terms of ‘systems research’, which can largely be ascribed to its routine inclusion of the plant stratum, i.e., through breeding thrusts for pest or disease resistance [72]. This added emphasis on HPR as a non-chemical preventative tactic in principle helps to advance sustainable forms of crop protection. However, improved crop genetics need to be consciously integrated with other agroecological or biodiversity-based tactics at field, farm, and landscape levels, i.e., as per the founding principles of IPM [30]. Doing so requires a stronger emphasis on public interest disciplines such as biological control, i.e., an area where the CGIAR has a markedly lower comparative advantage over national institutions. Therefore, while its current modus operandi may be appropriate to advance varietal improvement for a subset of staple crops, it is ill-suited to successfully harness a broader bundle of ecosystem services for sustainability [68]. Equally, the CGIAR is outpaced by national partners in areas such as taxonomic identification or molecular diagnostics. While pest-centric research in laboratory ‘microworlds’ is crucial for organismal identification or biosecurity, there are concerns that such research may narrow mindsets, slacken the flow of information, or hamper our understanding of ‘real-world’ ecological processes [73]. Though development cooperation could incrementally improve wholeness-oriented research, institutions such as the CGIAR are commended for actively investing in interdisciplinary, solution-oriented research, e.g., by methodically assessing the scope and societal reach of scientific thrusts [74,75]. We equally argue that the embryonic approach of hierarchical stratification constitutes a valuable approach to strategize science and guide institutional reform, being well suited to transcend nature–society dualisms and integrate resilience concepts [16,47].

Interdisciplinary ‘systems’ approaches are prone to close the gap between science and farm-level practice, but our work unveils how those are critically lagging in the Global South. For science to essentially mitigate pesticide abuse and improve agricultural sustainability, systems research is essential and close engagement with policymakers, extension or advisory services, and the private sector is warranted [33]. Each year, USD 58 billion is expended on pesticides globally [76], and the private sector invests USD 9 billion in agricultural input R&D at times when governments are wavering in their role as sponsors [42,77]. Agrochemical corporations reap the benefits of publicly funded science [2] but do not openly feature in the patchwork of institutions that aim to resolve pesticide-induced harm [78]. Equally, a small (though growing) fraction of the current funding of the CGIAR is allocated to agroecology or crop diversification compared with crop-centric activities, e.g., varietal improvement in core staple crops [79]. To complicate matters further, the diffusion of those agroecological practices is often hampered by a lack of awareness among farmers and government decision makers [80]. To remediate the above and provide renewed impetus for more sustainable forms of crop protection [81], reinvented institutional settings, paired with a re-routing of (public and private) funding streams, may be valuable. These ideally should be coupled with enabling policies to achieve farm-level transformations at scale [82], public–private coregulation [83], and institutional arrangements that nurture (cross-scale) interdisciplinary cooperation and collective action. Thus, reformed institutions, amended scientific trajectories, and modified incentive or reward structures can put pest management science more firmly on the systems track. Such novel arrangements may also help bridge the gap between what scientists are individually motivated to do and what they can accomplish together [84]. Those conditions appear indispensable for science to truly resolve the ‘grand challenge’ of feeding a swelling global population without jeopardizing Earth system resilience.

## Data Availability

All data underlying this manuscript are made available to readers in a public repository through https://doi.org/10.6084/m9.figshare.23514552 (accessed on 8 December 2023).

## Supplementary Materials

The following supporting information can be downloaded at: https://www.mdpi.com/article/10.3390/plants12244143/s1, Figure S1: Prisma flowchart; Figure S2: Principal crop (A) and pest foci (B) of pest management science in 65 developing countries; Figure S3: Institutional engagement in pest management science in different regions of the Global South over 2010-2020; Figure S4: Relative contribution of international development cooperation in pest management science for the 30 least developed countries; Table S1: Listing of countries that were included in the literature review, as organized by geographical sub-region.

## References

[1] Powell W.W., Snellman K. (2004). The knowledge economy. Annu. Rev. Sociol..

[2] Jefferson O.A., Jaffe A., Ashton D., Warren B., Koellhofer D., Dulleck U., Ballagh A., Moe J., DiCuccio M., Ward K. (2018). Mapping the global influence of published research on industry and innovation. Nat. Biotechnol..

[3] Park M., Leahey E., Funk R.J. (2023). Papers and patents are becoming less disruptive over time. Nature.

[4] King D.A. (2004). The scientific impact of nations. Nature.

[5] Miao L., Murray D., Jung W.-S., Larivière V., Sugimoto C.R., Ahn Y.-Y. (2022). The latent structure of global scientific development. Nat. Hum. Behav..

[6] Gomez C.J., Herman A.C., Parigi P. (2022). Leading countries in global science increasingly receive more citations than other countries doing similar research. Nat. Hum. Behav..

[7] Knox J., Hess T., Daccache A., Wheeler T. (2012). Climate change impacts on crop productivity in Africa and South Asia. Environ. Res. Lett..

[8] Savary S., Willocquet L., Pethybridge S.J., Esker P., McRoberts N., Nelson A. (2019). The global burden of pathogens and pests on major food crops. Nat. Ecol. Evol..

[9] DeFries R., Nagendra H. (2017). Ecosystem management as a wicked problem. Science.

[10] Acevedo M.F., Harvey D.R., Palis F.G. (2018). Food security and the environment: Interdisciplinary research to increase productivity while exercising environmental conservation. Glob. Food Secur..

[11] Yletyinen J., Brown P., Pech R., Hodges D., Hulme P.E., Malcolm T.F., Maseyk F.J.F., Peltzer D.A., Perry G.L.W., Richardson S.J. (2019). Understanding and Managing Social–Ecological Tipping Points in Primary Industries. BioScience.

[12] Willett W., Rockström J., Loken B., Springmann M., Lang T., Vermeulen S., Garnett T., Tilman D., DeClerck F., Wood A. (2019). Food in the Anthropocene: The EAT–Lancet Commission on healthy diets from sustainable food systems. Lancet.

[13] Rockström J., Edenhofer O., Gaertner J., DeClerck F. (2020). Planet-proofing the global food system. Nat. Food.

[14] Springmann M., Clark M., Mason-D’Croz D., Wiebe K., Bodirsky B.L., Lassaletta L., de Vries W., Vermeulen S.J., Herrero M., Carlson K.M. (2018). Options for keeping the food system within environmental limits. Nature.

[15] Tang F.H.M., Lenzen M., McBratney A., Maggi F. (2021). Risk of pesticide pollution at the global scale. Nat. Geosci..

[16] Nyström M., Jouffray J.B., Norström A.V., Crona B., Søgaard Jørgensen P., Carpenter S.R., Bodin Ö., Galaz V., Folke C. (2019). Anatomy and resilience of the global production ecosystem. Nature.

[17] Mahecha M.D., Bastos A., Bohn F.J., Eisenhauer N., Feilhauer H., Hartmann H., Hickler T., Kalesse-Los H., Migliavacca M., Otto F.E.L. (2022). Biodiversity loss and climate extremes—study the feedbacks. Nature.

[18] Smith M.R., Mueller N.D., Springmann M., Sulser T.B., Garibaldi L.A., Gerber J., Wiebe K., Myers S.S. (2022). Pollinator Deficits, Food Consumption, and Consequences for Human Health: A Modeling Study. Environ. Health Perspect..

[19] Wyckhuys K.A., Zhang W., Colmenarez Y.C., Simelton E., Sander B.O., Lu Y. (2022). Tritrophic defenses as a central pivot of low-emission, pest-suppressive farming systems. Curr. Opin. Environ. Sustain..

[20] Altieri M.A., Martin P.B., Lewis W.J. (1983). A quest for ecologically based pest management systems. Environ. Manag..

[21] Lewis W.J., Van Lenteren J.C., Phatak S.C., Tumlinson J.H. (1997). A total system approach to sustainable pest management. Proc. Natl. Acad. Sci. USA.

[22] Bromham L., Dinnage R., Hua X. (2016). Interdisciplinary research has consistently lower funding success. Nature.

[23] Fini R., Jourdan J., Perkmann M., Toschi L. (2023). A New Take on the Categorical Imperative: Gatekeeping, Boundary Maintenance, and Evaluation Penalties in Science. Organ. Sci..

[24] Bernhardt E.S., Rosi E.J., Gessner M.O. (2017). Synthetic chemicals as agents of global change. Front. Ecol. Environ..

[25] Carson R. (1962). Silent Spring.

[26] Savary S., Akter S., Almekinders C., Harris J., Korsten L., Rötter R., Waddington S., Watson D. (2020). Mapping disruption and resilience mechanisms in food systems. Food Secur..

[27] Dainese M., Martin E.A., Aizen M.A., Albrecht M., Bartomeus I., Bommarco R., Carvalheiro L.G., Chaplin-Kramer R., Gagic V., Garibaldi L.A. (2019). A global synthesis reveals biodiversity-mediated benefits for crop production. Sci. Adv..

[28] Shattuck A., Werner M., Mempel F., Dunivin Z., Galt R. (2023). Global pesticide use and trade database (GloPUT): New estimates show pesticide use trends in low-income countries substantially underestimated. Glob. Environ. Chang..

[29] Deutsch C.A., Tewksbury J.J., Tigchelaar M., Battisti D.S., Merrill S.C., Huey R.B., Naylor R.L. (2018). Increase in crop losses to insect pests in a warming climate. Science.

[30] Deguine J.-P., Aubertot J.-N., Flor R.J., Lescourret F., Wyckhuys K.A., Ratnadass A. (2021). Integrated pest management: Good intentions, hard realities. A review. Agron. Sustain. Dev..

[31] Bommarco R., Kleijn D., Potts S.G. (2013). Ecological intensification: Harnessing ecosystem services for food security. Trends Ecol. Evol..

[32] Pretty J. (2020). New opportunities for the redesign of agricultural and food systems. Agric. Hum. Values.

[33] Wyckhuys K.A., Zou Y., Wanger T.C., Zhou W., Gc Y.D., Lu Y. (2022). Agro-ecology science relates to economic development but not global pesticide pollution. J. Environ. Manag..

[34] González-Chang M., Wratten S.D., Shields M.W., Costanza R., Dainese M., Gurr G.M., Johnson J., Karp D.S., Ketelaar J.W., Nboyine J. (2020). Understanding the pathways from biodiversity to agro-ecological outcomes: A new, interactive approach. Agric. Ecosyst. Environ..

[35] Messing R., Brodeur J. (2018). Current challenges to the implementation of classical biological control. BioControl.

[36] Walker B., Barrett S., Polasky S., Galaz V., Folke C., Engström G., Ackerman F., Arrow K., Carpenter S., Chopra K. (2009). Looming Global-Scale Failures and Missing Institutions. Science.

[37] Deguine J.P., Aubertot J.N., Bellon S., Côte F.X., Lauri P.E.E., Lescourret F., Ratnadass A., Scopel E., Andrieu N., Bàrberi P. (2023). Agroecological crop protection for sustainable agriculture. Adv. Agron..

[38] Mansfield B., Werner M., Berndt C., Shattuck A., Galt R., Williams B., Argüelles L., Barri F.R., Ishii M., Kunin J. (2023). A new critical social science research agenda on pesticides. Agric. Hum. Values.

[39] Alrøe H.F., Kristensen E.S. (2002). Towards a systemic research methodology in agriculture: Rethinking the role of values in science. Agric. Hum. Values.

[40] Vanloqueren G., Baret P.V. (2009). How agricultural research systems shape a technological regime that develops genetic engineering but locks out agroecological innovations. Res. Policy.

[41] Vanbergen A.J., Aizen M.A., Cordeau S., Garibaldi L.A., Garratt M.P., Kovács-Hostyánszki A., Lecuyer L., Ngo H.T., Potts S.G., Settele J. (2020). Transformation of agricultural landscapes in the Anthropocene: Nature’s contributions to people, agriculture and food security. Advances in Ecological Research.

[42] Clapp J. (2021). The problem with growing corporate concentration and power in the global food system. Nat. Food.

[43] Warner K.D., Daane K.M., Getz C.M., Maurano S.P., Calderon S., Powers K.A. (2011). The decline of public interest agricultural science and the dubious future of crop biological control in California. Agric. Hum. Values.

[44] Mastrángelo M.E., Pérez-Harguindeguy N., Enrico L., Bennett E., Lavorel S., Cumming G.S., Abeygunawardane D., Amarilla L.D., Burkhard B., Egoh B.N. (2019). Key knowledge gaps to achieve global sustainability goals. Nat. Sustain..

[45] Andersen A.D., Steen M., Mäkitie T., Hanson J., Thune T.M., Soppe B. (2020). The role of inter-sectoral dynamics in sustainability transitions: A comment on the transitions research agenda. Environ. Innov. Soc. Transit..

[46] Tscharntke T., Grass I., Wanger T.C., Westphal C., Batáry P. (2021). Beyond organic farming–harnessing biodiversity-friendly landscapes. Trends Ecol. Evol..

[47] Jansen K. (2009). Implicit Sociology, Interdisciplinarity and Systems Theories in Agricultural Science. Sociol. Rural..

[48] Wyckhuys K.A.G., Tang F.H.M., Hadi B.A.R. (2023). Pest management science often disregards farming system complexities. Commun. Earth Environ..

[49] Naranjo S.E., Hellmich R.L., Romeis J., Shelton A.M., Velez A.M., Kogan M., Heinrichs E. (2020). The role and use of genetically engineered insect-resistant crops in IPM systems. Integrated Management of Insect Pests: Current and Future Developments.

[50] Cavacini A. (2016). Recent trends in Middle Eastern scientific production. Scientometrics.

[51] May R.M. (1997). The Scientific Wealth of Nations. Science.

[52] Courtioux P., Métivier F., Rebérioux A. (2022). Nations ranking in scientific competition: Countries get what they paid for. Econ. Model..

[53] Waddington H., Snilstveit B., Hombrados J., Vojtkova M., Phillips D., Davies P., White H. (2014). Farmer Field Schools for Improving Farming Practices and Farmer Outcomes: A Systematic Review. Campbell Syst. Rev..

[54] Wyckhuys K.A.G., Lu Y., Zhou W., Cock M.J.W., Naranjo S.E., Fereti A., Williams F.E., Furlong M.J. (2020). Ecological pest control fortifies agricultural growth in Asia–Pacific economies. Nat. Ecol. Evol..

[55] Godtland E.M., Sadoulet E., de Janvry A., Murgai R., Ortiz O. (2004). The Impact of Farmer Field Schools on Knowledge and Productivity: A Study of Potato Farmers in the Peruvian Andes. Econ. Dev. Cult. Chang..

[56] Keeler B.L., Chaplin R.E., Guerry A.D., Addison P.F.E., Bettigole C., Burke I.C., Gentry B., Chambliss L., Young C., Travis A.J. (2017). Society Is Ready for a New Kind of Science—Is Academia?. Bioscience.

[57] Levidow L., Pimbert M., Vanloqueren G. (2014). Agroecological research: Conforming—Or transforming the dominant agro-food regime?. Agroecol. Sustain. Food Syst..

[58] Pretty J., Benton T.G., Bharucha Z.P., Dicks L.V., Flora C.B., Godfray H.C.J., Goulson D., Hartley S., Lampkin N., Morris C. (2018). Global assessment of agricultural system redesign for sustainable intensification. Nat. Sustain..

[59] Brodeur J., Abram P.K., Heimpel G.E., Messing R.H. (2018). Trends in biological control: Public interest, international networking and research direction. BioControl.

[60] Lavelle P., Mathieu J., Spain A., Brown G., Fragoso C., Lapied E., De Aquino A., Barois I., Barrios E., Barros M.E. (2022). Soil macroinvertebrate communities: A world-wide assessment. Glob. Ecol. Biogeogr..

[61] Coll M., Wajnberg E., Coll M., Wajnberg E. (2017). Environmental pest management: A call to shift from a pest- centric to a system-centric approach. Environmental Pest Management: Challenges for Agronomists, Ecologists, Economists and Policymakers.

[62] Dentzman K. (2022). Academics and the ‘easy button’: Lessons from pesticide resistance management. Agric. Hum. Values.

[63] Rosa-Schleich J., Loos J., Mußhoff O., Tscharntke T. (2019). Ecological-economic trade-offs of Diversified Farming Systems—A review. Ecol. Econ..

[64] Tamburini G., Bommarco R., Wanger T.C., Kremen C., van der Heijden M.G.A., Liebman M., Hallin S. (2020). Agricultural diversification promotes multiple ecosystem services without compromising yield. Sci. Adv..

[65] Wratten S.D., Hofmans M., Thomsen S., Williams P., Groves G., Eason C., Greer J. Measuring sustainability in agricultural systems. Proceedings of the New Zealand Plant Protection Conference.

[66] Olson R. (1990). Science Deified and Science Defied.

[67] Woolston C. (2023). How to measure the societal impact of science. Nature.

[68] Cash D.W., Clark W.C., Alcock F., Dickson N.M., Eckley N., Guston D.H., Jäger J., Mitchell R.B. (2003). Knowledge systems for sustainable development. Proc. Natl. Acad. Sci. USA.

[69] Dentzman K. (2022). Governance of emerging pests and pathogens in production landscapes: Pesticide resistance and collaborative governance. Curr. Opin. Environ. Sustain..

[70] Altieri M.A., Francis C.A., Van Schoonhoven A., Doll J.D. (1978). A review of insect prevalence in maize (*Zea mays* L.) and bean (*Phaseolus vulgaris* L.) polycultural systems. Field Crop. Res..

[71] Herren H.R., Neuenschwander P. (1991). Biological control of cassava pests in Africa. Annu. Rev. Entomol..

[72] Kholová J., Urban M.O., Cock J., Arcos J., Arnaud E., Aytekin D., Azevedo V., Barnes A.P., Ceccarelli S., Chavarriaga P. (2021). In pursuit of a better world: Crop improvement and the CGIAR. J. Exp. Bot..

[73] Rosenheim J.A., Coll M. (2008). Pest-Centric versus Process-Centric Research Approaches in Agricultural Entomology. Am. Èntomol..

[74] Petsakos A., Prager S.D., Gonzalez C.E., Gama A.C., Sulser T.B., Gbegbelegbe S., Kikulwe E.M., Hareau G. (2019). Understanding the consequences of changes in the production frontiers for roots, tubers and bananas. Glob. Food Secur..

[75] Carneiro B., Resce G., Sapkota T.B. (2022). Digital artifacts reveal development and diffusion of climate research. Sci. Rep..

[76] Chaplin-Kramer R., O’Rourke M., Schellhorn N., Zhang W., Robinson B.E., Gratton C., Rosenheim J.A., Tscharntke T., Karp D.S. (2019). Measuring What Matters: Actionable Information for Conservation Biocontrol in Multifunctional Landscapes. Front. Sustain. Food Syst..

[77] Beintema N.M., Stads G.J. (2019). Measuring agricultural research investments: A revised global picture. Gates Open Res..

[78] Kinniburgh F., Selin H., Selin N.E., Schreurs M. (2022). When private governance impedes multilateralism: The case of international pesticide governance. Regul. Gov..

[79] Ickowitz A., McMullin S., Rosenstock T., Dawson I., Rowland D., Powell B., Mausch K., Djoudi H., Sunderland T., Nurhasan M. (2022). Transforming food systems with trees and forests. Lancet Planet. Health.

[80] Agboka K.M., Tonnang H.E., Abdel-Rahman E.M., Odindi J., Mutanga O., Niassy S. (2022). Data-driven artificial intelligence (AI) algorithms for modelling potential maize yield under maize–legume farming systems in East Africa. Agronomy.

[81] Gautam M., Laborde D., Mamun A., Martin W., Pineiro V., Vos R. (2022). Repurposing Agricultural Policies and Support: Options to Transform Agriculture and Food Systems to Better Serve the Health of People, Economies, and the Planet.

[82] Möhring N., Ingold K., Kudsk P., Martin-Laurent F., Niggli U., Siegrist M., Studer B., Walter A., Finger R. (2020). Pathways for advancing pesticide policies. Nat. Food.

[83] Kolcava D., Rudolph L., Bernauer T. (2021). Citizen preferences on private-public co-regulation in environmental governance: Evidence from Switzerland. Glob. Environ. Chang..

[84] Schelling T.C. (1978). Micromotives and Macrobehavior.

