# Supplementary material for: Institutional Context of Pest Management Science in the Global South
Source: Plants (Basel). 2023 Dec 12;12(24):4143. doi: 10.3390/plants12244143 (PMC10747170; doi:10.3390/plants12244143)
Supplement: Supplementary file 1 [file plants-12-04143-s001.zip › plants-2681395-supplementary.pdf]

plants MDPI

Supplementary Materials

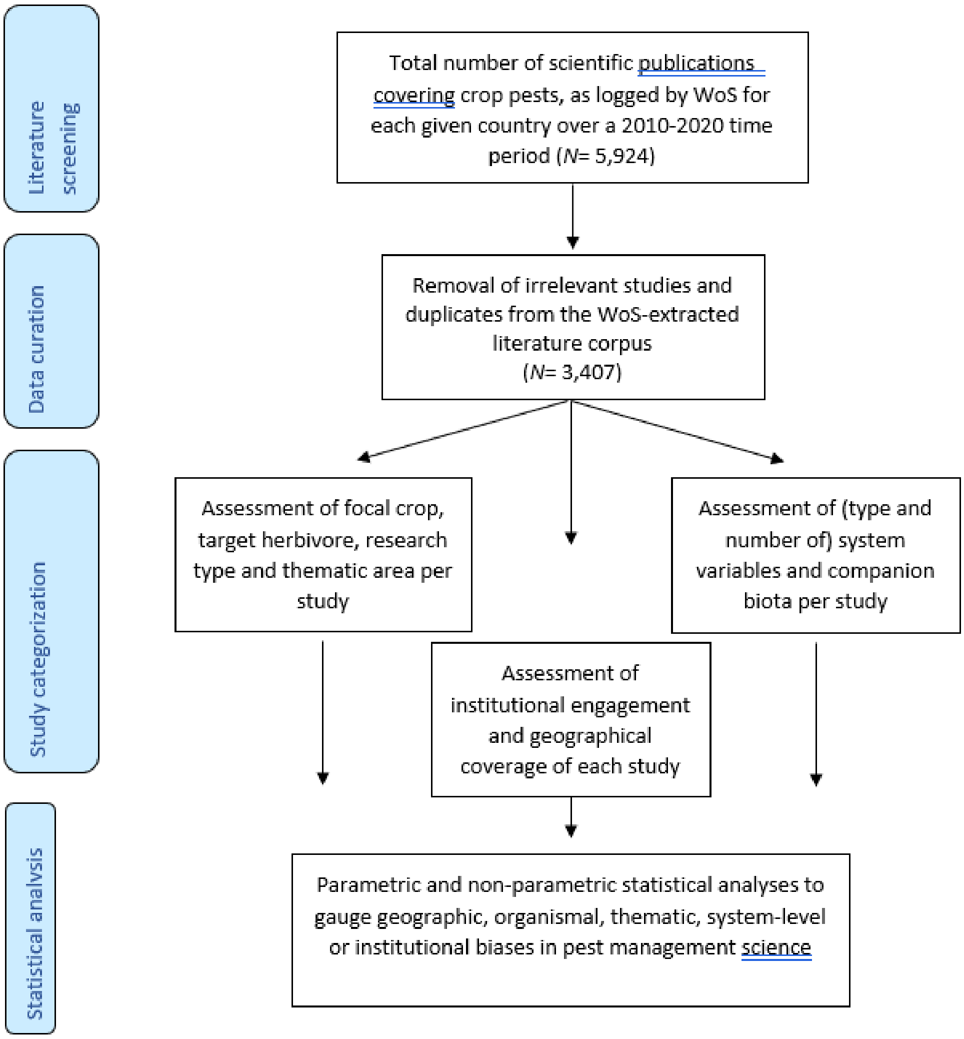

Figure S1. Prisma flowchart summarizing how bibliometric methods and literature analyses were used to characterize target herbivore, research type, thematic focus, system-level variables and companion biota of pest management science in 65 developing countries; WoS: Web of Science.

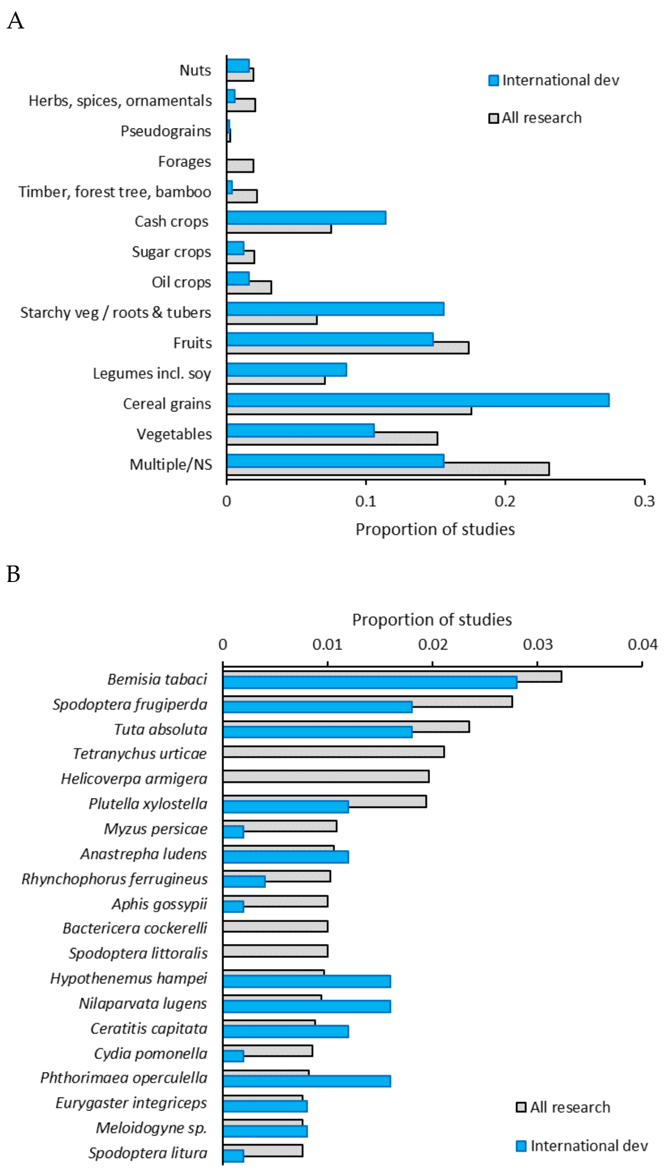

Figure S2. Principal crop (A) and pest foci (B) of pest management science in 65 developing countries. Patterns are plotted for 3,407 peer-reviewed publications over 2010-2020. In each panel, a distinction is made between all published research and studies with involvement of international development partners. Panel A organizes data in 14 crop categories, in which NS refers to 'non-specified'. Fruits comprise both annual and perennial fruits e.g., date palm. Oil crops include sunflower, oilseed and olive, while pseudo-grains cover amaranth, buckwheat, quinoa and chia. Panel B shows the proportion of studies that cover either of the 20 most researched pest taxa. .

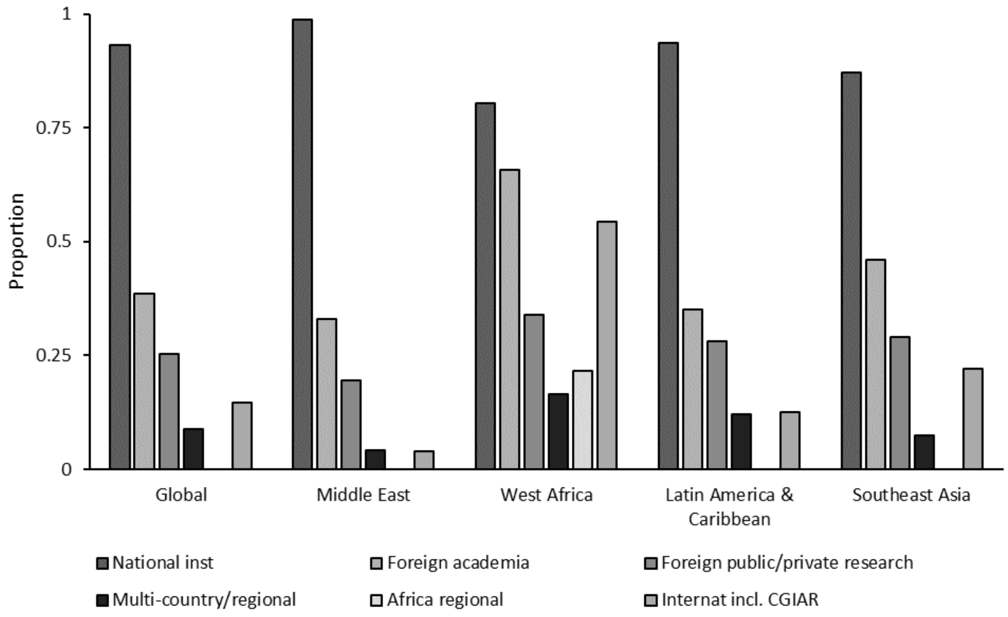

Figure S3. Institutional engagement in pest management science in different regions of the Global South over 2010-2020. Bar charts depict global and sub-regional patterns in the extent of involvement of national institutions, foreign academia and foreign public or private research institutions. Data are extracted from a total of 3,407 peer-reviewed international publications, covering 65 countries. Per geography, the proportion of studies with multi-country or regional cooperation or support from international development partners e.g., CGIAR is also plotted. For West Africa, regional cooperation at the continental level (i.e., Africa) is equally depicted.

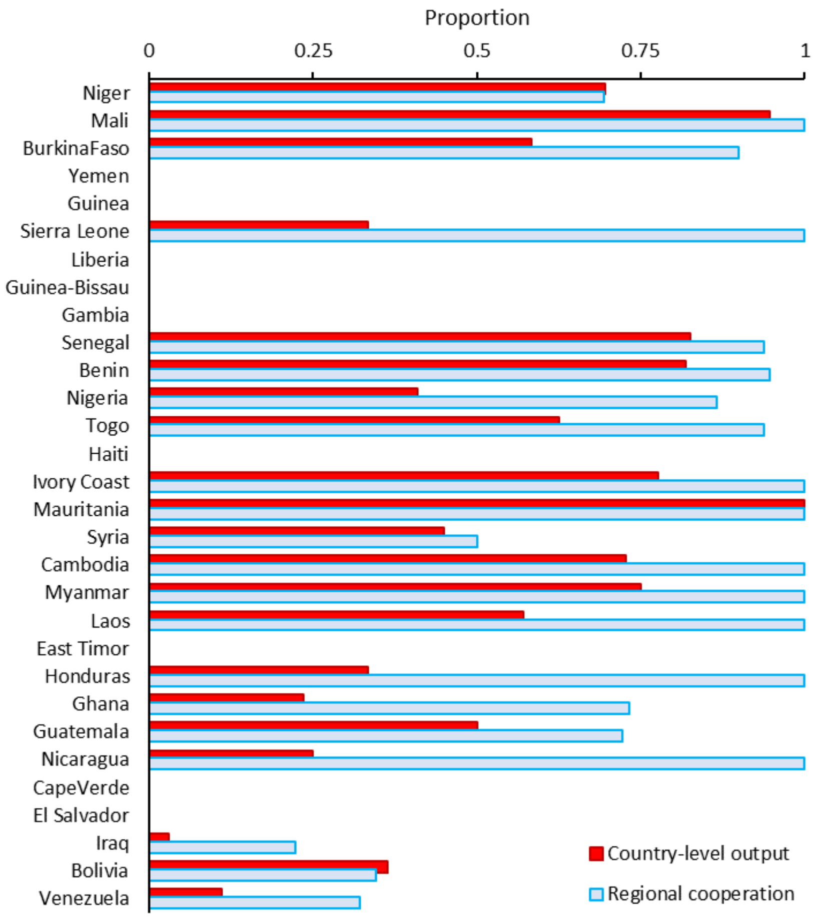

Figure S4. Relative contribution of international development cooperation in pest management science for the 30 least developed countries. Data refer to country-level publication output over 2010-2020. Per country, we plot the proportion of country-level research output (red) and regional cooperative studies (blue) in which development partners feature. Patterns are shown for countries within the 4 target sub-regions with human development index (HDI) rank equal or above 118. Countries are ranked from least (top) to most developed (bottom). .

Table S1. Listing of countries that were included in the literature review, as organized by geographical sub-region.

Middle East	West Africa	Latin America	Southeast Asia
Bahrain	Benin	Argentina	Brunei
Cyprus	Burkina Faso	Bolivia	Cambodia
Egypt	Cape Verde	Brazil	East Timor
Iran	Gambia	Chile	Indonesia
Iraq	Ghana	Colombia	Laos
Israel	Guinea	Costa Rica	Malaysia
Jordan	Guinea-Bissau	Cuba	Myanmar
Kuwait	Ivory Coast	Dominican Republic	Philippines
Lebanon	Liberia	Ecuador	Singapore
Oman	Mali	El Salvador	Thailand
Palestine	Mauritania	Guatemala	Vietnam
Qatar	Niger	Haiti	
Saudi Arabia	Nigeria	Honduras	
Syria	Senegal	Mexico	
Turkey	Sierra Leone	Nicaragua	
UAE	Togo	Panama	
Yemen		Paraguay	
		Peru	
		Uruguay	
		Venezuela	
